# The Stem Cell Connection of Pituitary Tumors

**DOI:** 10.3389/fendo.2017.00339

**Published:** 2017-12-04

**Authors:** Hugo Vankelecom, Heleen Roose

**Affiliations:** ^1^Department of Development and Regeneration, Cluster of Stem Cell and Developmental Biology, Unit of Stem Cell Research, University of Leuven (KU Leuven), Leuven, Belgium

**Keywords:** pituitary tumor, stem cells, tumor stem cells, SOX2, NOTCH, WNT, epithelial–mesenchymal transition, Hippo

## Abstract

Tumors in the pituitary gland are typically benign but cause serious morbidity due to compression of neighboring structures and hormonal disruptions. Overall, therapy efficiency remains suboptimal with negative impact on health and comfort of life, including considerable risk of therapy resistance and tumor recurrence. To date, little is known on the pathogenesis of pituitary tumors. Stem cells may represent important forces in this process. The pituitary tumors may contain a driving tumor stem cell population while the resident tissue stem cells may be directly or indirectly linked to tumor development and growth. Here, we will briefly summarize recent studies that afforded a glance behind the scenes of this stem cell connection. A better knowledge of the mechanisms underlying pituitary tumorigenesis is essential to identify more efficacious treatment modalities and improve clinical management.

## Introduction

Pituitary tumors represent the most common intracranial tumor type (1, 2). Although typically benign with only very rare metastasis, these tumors can cause significant morbidity including severe headache, visual defects, and panhypopituitarism due to compression of neighboring structures, as well as major endocrine disturbances through hypersecretion of hormones, responsible for clinical syndromes that are fatal if not appropriately treated. Classically, the tumors arising in the anterior pituitary (AP), the major endocrine lobe of the gland, are subdivided in prolactinomas that oversecrete prolactin (PRL); somatotropinomas that over-produce growth hormone (GH); corticotropinomas producing excessive amounts of adrenocorticotropic hormone (ACTH); thyrotropinomas that hyper-secrete thyroid-stimulating hormone (TSH); gonadotropinomas that produce luteinizing hormone (LH) and/or follicle-stimulating hormone (FSH); pluri-hormonal adenomas producing more than one hormone; and non-functional pituitary adenomas (NFPA) which do not detectably secrete hormones (1, 2). In addition, craniopharyngiomas occur in the pituitary (sellar/parasellar) region, categorized as adamantinomatous craniopharyngioma (ACP) mostly encountered in children, and papillary craniopharyngioma (PCP) mostly diagnosed in elderly patients (3–5). Also blastomas and, extremely rarely, carcinomas develop in the pituitary, whereas pituicytomas, oncocytomas and ependymomas can arise in the posterior (neural) lobe of the gland (2).

Despite sizeable benefit of current treatment procedures, including transsphenoidal resection, irradiation, and medicines, therapy efficiency remains suboptimal with important negative impact on health and quality of life (1, 6–8). Moreover, therapy resistance as well as recurrence of the tumor is often encountered; relapse is observed in more than 50% of patients in case of pituitary adenomas with invasive behavior (1, 2).

As of today, little is known on the molecular and cellular underpinnings of pituitary tumorigenesis (1, 2, 6). A clearer understanding of the underlying mechanisms is fundamentally and clinically important.

## The Stem Cell Connection of Pituitary Tumors

In this Perspective, we will discuss the potential stem cell connection of pituitary tumors from three angles. First, we briefly describe recent findings pointing to the presence of putative “tumor stem cells.” Then, we give a short overview of what is known on the behavior of the tissue’s own stem cells during tumorigenesis in the gland, thereby revealing possible links. Finally, we bring together findings on pathways generally known to regulate (tumor) stem cells.

## Tumor Stem Cell Candidates

During the last decades, cancer stem cells (CSC) were identified in various types of cancer (9–11). CSC are considered to represent the tumor cell subpopulation that drives tumor growth, sustenance, local invasion and/or metastasis, as well as resistance to therapy and subsequent re-growth (9–12). The experimental hallmark of CSC is tumorigenic dominance, i.e., they regrow the tumor with higher efficiency than the rest of the tumor cells. Thus, CSC represent an interesting target to better understand and treat the particular cancer. CSC possess the stem cell characteristics of self-renewal and differentiation potential to (re-)grow the heterogeneous tumor. Their origin may vary according to cancer type: mature tissue cells may de-differentiate and regain stemness properties, or resident stem cells may adopt aberrant (growth) characteristics to become CSC (13–18). Whether pituitary tumors, or at least particular subtypes, adhere to the CSC model has been the focus of a number of recent studies. Because pituitary tumors are generally not considered as cancer, we will further not use the term CSC but designate this tumor-driving population as “tumor stem cells” (TSC). Extensive reviews have been published on this topic before (19–24). Here, we will briefly summarize and discuss the more recent findings.

Candidate TSC populations have been identified in pituitary tumors using several different approaches (Table 1). Human pituitary adenomas (as reported for one somatotropinoma and one null cell adenoma) were found to contain cells that generate sphere-like structures in culture (an *in vitro* property of stem cells) displaying expression of some general stemness markers (like nestin and CD133) and possessing some—although limited—differentiation capacity (25). Another study also identified pituitary adenoma cells with CD133 expression, and self-renewal and (limited) differentiation capacity (as analyzed in mainly somatotropinomas and NFPA) (26). However, these cells were sensitive to the anti-proliferative effect of a dopamine/somatostatin chimeric agonist which is uncharacteristic for TSC which should be therapy-resistant (Table 1). Manoranjan et al. (27) identified a CD15^+^ cell subpopulation in human pituitary adenomas (of different histotypes, and in particular somatotropinomas and NFPA). These cells had higher sphere-forming capacity and elevated *SOX2* gene expression. An earlier study already reported elevated gene and protein levels of SOX2 in a putative TSC population, as identified by side population (SP) efflux capacity for Hoechst dye (analyzed in multiple tumor histotypes, and in particular somatotropinomas and NFPA) (28). Efficient efflux capacity is considered one of the mechanisms underlying TSC resistance to anti-cancer drugs. The pituitary tumor SP was found enriched in cells with pronounced expression of tumor stemness markers (such as SOX2 and the chemokine C-X-C motif receptor 4, CXCR4) and of stem cell-associated signaling pathways [such as epithelial–mesenchymal transition, (EMT)]. Moreover, the SP contained cells possessing self-renewal competence as shown by serial sphere formation *in vitro*, and tumorigenic dominance as examined by subcutaneous xenotransplantation in immunodeficient mice (the latter analyzed using the mouse pituitary tumor AtT20 cell line) (28) (Table 1). The SP cells also showed higher resistance to temozolomide, a chemotherapeutic drug used to treat resistant pituitary tumors (Roose and Vankelecom, unpublished observations). In addition, CXCR4 signaling appeared to be functionally involved in pituitary tumorigenesis; inhibition of the pathway reduced the size of tumors growing subcutaneously from AtT20 cells in immunodeficient mice. Moreover, CXCR4 inhibition also decreased EMT-associated cell motility *in vitro* as analyzed using the scratch assay (28). The SP of benign human pituitary tumors showed some tantalizing expression differences from the candidate TSC (SP) isolated from human malignant cancer samples [melanoma and pancreatic cancer (29, 30)]; such as upregulated expression of senescence markers (e.g., *p21, BCL2, IL6, IL8*; Vankelecom, unpublished observations). Senescence may explain why pituitary tumors typically remain benign (31). Increased expression of SOX2 protein was also found in the tumor-bearing pituitary of the dopamine receptor D2 knock-out (*Drd2*^−/−^) mouse model (28). Female *Drd2*^−/−^ mice develop prolactinomas and it was observed that the *Drd2*^−/−^ pituitaries have a greater proportion of SP cells and of proliferating (Ki67^+^) SOX2^+^ cells than control pituitary tissue (28). The latter findings may suggest different possibilities, including (i) a lineal relationship between the pituitary resident stem cells (as marked by SOX2 expression and SP efflux capacity) and the putative TSC, or in other words, TSC directly originating from the activated or deregulated pituitary stem cells; and/or (ii) activation and proliferation of the resident stem cells (without giving rise to the TSC) when tumorigenesis is occurring in the gland (see further below).

**Table 1:** Stem cell connections of pituitary tumors: overview and current limitations.

**Candidate TSC**	**Stemness marker/pathway**	**Functional TSC property**	**Human tumor histotype**	**Reference**	**Current limitations**
	Nestin CD133	– Sphere-like structures – Limited differentiation	GH^+^, NFPA	(25)	– Limited number of adenomas (2) analyzed – Convincing *in vivo* xenotransplantation from human pituitary tumors still missing

	CD133	– Self-renewal – Limited differentiation	GH^+^, NFPA	(26)	– Failure to resist therapy *–In vivo* xenotransplantation from human pituitary tumors still missing

	CD15 SOX2	Sphere-forming capacity	Multiple types	(27)	*In vivo* xenotransplantation from human pituitary tumors still missing

	SOX2 CXCR4 EMT	– SP phenotype – Sphere-forming capacity – Self-renewal – *In vivo* tumorigenic dominance (SP from AtT20 cell line)	Multiple types (including PRL^+^ from mouse *Drd2*^−^*^/^*^−^ pituitary)	(28)	*In vivo* xenotransplantation from human pituitary tumors still missing
		– Resistance to temozolomide		Unpublished	
		– Upregulation of senescence markers		Unpublished	

**Link with resident stem cells**	**Stem/progenitor cell marker (model/species)**	**Observation**	**Tumor type**	**Reference**	**Current limitations**

	HESX1, SOX2 (*Hesx1/mutant* β*-catenin* and *Sox2/mutant* β*-catenin* mouse)	Stem cells as paracrine inducer and stimulator of tumor growth	ACP-replicating	(3, 4, 32)	Unequivocal demonstration of the need for paracrine signaling from the stem cells still missing

	SOX2, SOX9, OCT4, KLF4 (human)	Expression	ACP and PCP	(5, 33)	Circumstantial evidence

	SOX2 (human) HESX1 (*Hesx1/mutant Braf* or *mutant Kras* mouse)	Major proliferative cell population (⇑tumor-driving?) Increased proliferation and decreased differentiation of SOX2^+^ cells	PCP	(34)	Stem cell lineage tracing still missing (using mouse models) – No tumor growth at perinatal age of death – If tumor growth, stem cell lineage tracing needed
				(34)	

	Nestin, SOX2 (*Rb^+^^/^^−^* mouse)	Nestin^+^-traced and SOX2^+^ cells in proximity of pituitary tumors (⇑paracrine role?)	IL	(35)	Stem cell lineage tracing still missing

	SOX2 (*p27*^−^*^/^*^−^ mouse)	Expanded SOX2^+^ zone in pituitary	IL	(36)	Stem cell lineage tracing still missing

	αGSU (α*Gsu/Pttg* mouse)	Pituitary tumor development	Uni- (LH) and pluri-hormonal (LH, TSH, GH) tumors	(37)	Stem cell examination and lineage tracing still missing

	PROP1, αGSU (α*Gsu/Prop1* mouse)	PROP1-overexpressing cells in proximity of pituitary tumors (⇑paracrine role?)	Multiple types	(38, 39)	Stem cell lineage tracing still missing

	BMI1 (*Gfap/Bmi1* mouse)		ACTH (IL and AP)	(40)	Stem cell lineage tracing still missing

	SOX2 (SOX2^+^ lineage tracing in *Drd2^−/−^* mouse)	No major co-localization of PRL and SOX2 (⇑no direct link, but paracrine role?)	PRL	Unpublished (Figure 1)	Support for paracrine role still missing

**Stem cell regulatory pathways (as found in stem cells and candidate TSC)**	**Stemness pathway**	**Observation**	**Human tumor histotype**	**Reference**	**Current limitations**

	NOTCH	Upregulated genes in SP	GH, NFPA	(28)	Support for functional role still missing

		Upregulated genes in spheres and CD133^+^ cells	GH, NFPA	(25, 26)	Support for functional role still missing

	WNT	Upregulated genes in SP	GH, NFPA	(28)	Support for functional role still missing

		Upregulated genes in SOX2^+^ cells of ACP mouse	ACP-replicating	(3, 4, 32)	Evidence for functional role still missing

	EMT	– Upregulated mesenchymal genes in SP – Downregulated epithelial genes in SP	GH, NFPA	(28)	Support for functional role still missing

	SHH	Upregulated genes in SOX2^+^ cells of ACP mouse	ACP-replicating	(4, 32)	Support for functional role still missing

	Hippo	Upregulated genes in SP	GH, NFPA	(28)	Support for functional role still missing

	EGF/FGF	Upregulated genes (encoding the growth factor receptors) in SP	GH, NFPA	(28)	Support for functional role still missing

Taken together, particular TSC candidates in pituitary tumors have been advanced, but definitive evidence of self-renewable and serially transplantable tumor-initiating cells that regrow the original tumor with high(-er) efficiency, has not yet been provided (Table 1). It should be realized that important barriers hinder the identification of *bona fide* pituitary tumor-initiating cells using the “golden” *in vivo* xenotransplantation test. Pituitary adenomas are generally benign and quiescent (i.e., low proliferative phenotype) predicting a poor *in vivo* growth propensity. Moreover, being from benign tumors, TSC may need to be implanted in their natural habitat to enable propagation; however, it is technically very difficult to implant cells orthotopically in the pituitary region. Nevertheless, conclusive identification and characterization of an unambiguous TSC population would significantly deepen our knowledge on the as yet poorly understood mechanisms of pituitary tumor pathogenesis and unveil potential novel targets for therapeutic interventions.

## Relation Between Pituitary Stem Cells and Tumorigenesis

What is the position of the pituitary’s own resident stem cells in the process of tumorigenesis in the gland? Are these stem cells directly involved in generating and growing the pituitary tumors (thus in generating the TSC), or do they become activated because of the threatening tumorigenic event in their tissue? Recent studies revealed that pituitary stem cells are activated in other kinds of jeopardizing events occurring in the pituitary like cell-ablation injury (41–43). Here, we briefly summarize studies that lifted some tip on the functional position of pituitary stem/progenitor cells in tumor formation in the gland (Table 1).

ACP is often accompanied by gene mutations in the WNT signaling mediator β-catenin that prevent its degradation, thereby allowing continuous β-catenin/WNT signaling to the nucleus (3–5). In a transgenic mouse model of ACP, targeted expression of degradation-resistant β-catenin in early-embryonic pituitary progenitor (HESX1^+^) cells or in SOX2^+^ pituitary stem cells induced a transient proliferative response in the SOX2^+^ cell population (3, 4). SOX2^+^ lineage tracing (allowing to follow the SOX2^+^ cells as well as their progeny over time) showed that the mutant β-catenin-expressing SOX2^+^ cells did not directly give rise to the ACP tumor, but induced the tumor and promoted the proliferation of the surrounding (tumor) cells (Table 1), most likely *via* the production of paracrine factors belonging to key (stemness) pathways (such as WNT; sonic hedgehog, SHH; fibroblast growth factor, FGF; bone-morphogenetic protein, BMP) (3, 32). Interestingly, tumor development was not observed when mutant β-catenin was expressed in committed or differentiated (GH^+^ and PRL^+^) pituitary cells, indicating the importance of the (mutated) stem cells as paracrine signaling center (3). Expression of the pituitary stem cell markers SOX2, SOX9, OCT4, and KLF4, as identified in normal human pituitaries, was also observed in human ACP (and PCP) samples (5, 33) (Table 1). A recent study showed a sustained proliferative phenotype of SOX2^+^ cells in human PCP (known to predominantly harbor BRAF mutations), and advanced the hypothesis that these cells may be driving the growth of the tumor because they represent the major proliferative cell population within the tumor (34). This assumption as well as the nature of the link (direct or indirect) awaits further investigation, for instance, using the *Braf* and *Kras* mutant mouse models described in the same study (34). However, it has first to be shown that pituitary tumors indeed develop in the latter models (in analogy with BRAF mutations in humans). *Braf* and *Kras* mutant mice die perinatally and do not display pituitary tumors (yet) at this early age (34) (Table 1). Therefore, these mouse models need first to be refined, for instance, by applying a conditional system allowing to mutate the genes after birth. Another study reported the presence of nestin^+^ lineage-derived and SOX2^+^ cells in the close proximity of tumors developing in the pituitary intermediate lobe (IL) of mutant retinoblastoma (*Rb^+/−^*) mice (35), thus suggesting not a direct relationship but a paracrine role of the stem cells in this particular tumor type. Genetic deletion of the tumor suppressor p27 in mice also results in pituitary IL tumors, accompanied by increased thickness of the SOX2^+^ stem cell zone (36) (Table 1). The occurrence of pituitary tumors was markedly reduced in *p27^−/−^* mice when one SOX2 allele was inactivated, suggesting a role for SOX2 as oncogene. By contrast, SOX2 haploinsufficiency with decreased functionality of the mutant protein may also be correlated with pituitary tumorigenesis (as observed in human patients) (44), suggesting that SOX2 may also act as tumor suppressor.

**Figure 1:**
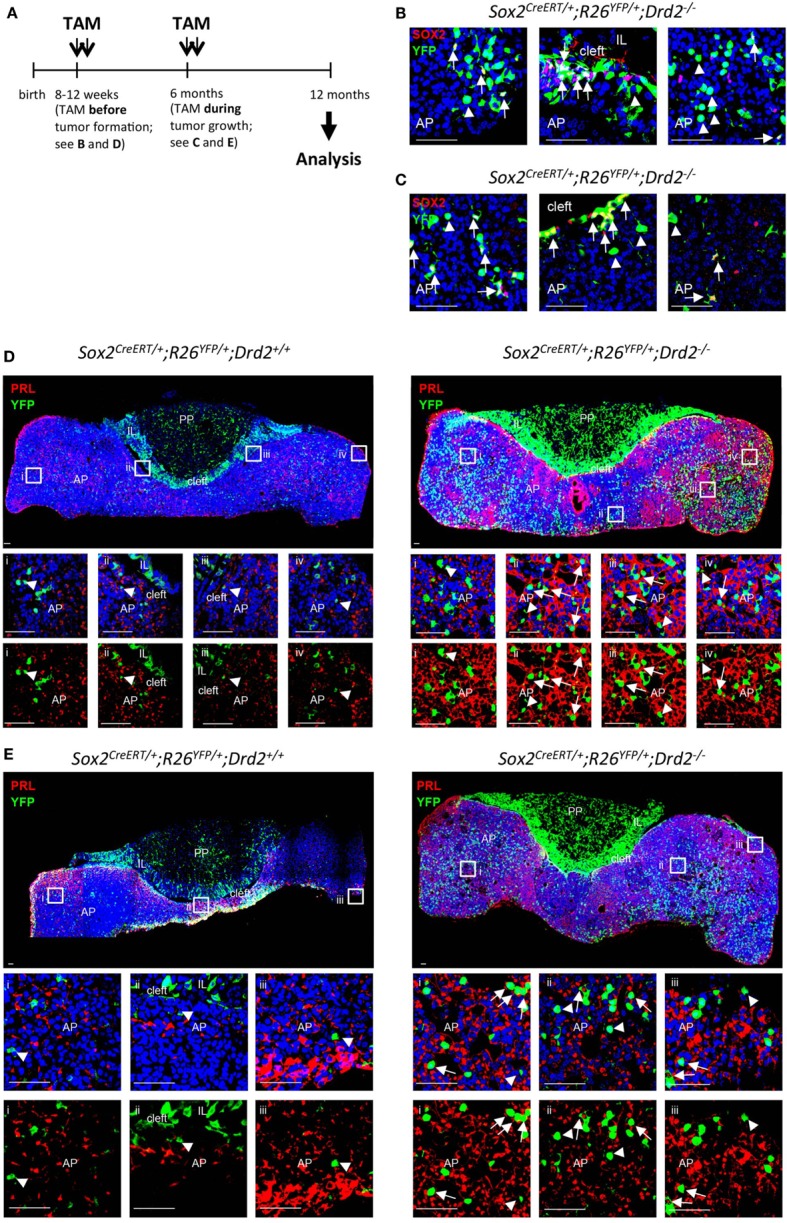
SOX2^+^ lineage tracing in the prolactinoma-developing Drd2^−/−^ mouse model. **(A)** Time schedule of tamoxifen (TAM) induction of SOX2^+^ lineage tracing. TAM (0.15 mg/g body weight/day) was injected for two consecutive days at the age of 8–12 weeks (i.e., *before* tumor formation) or at the age of 6 months (i.e., *during* tumor development and growth). Immunofluorescence analysis was done at 12 months of age. **(B,C)** Immunofluorescence staining for SOX2 (red) and YFP (green) in pituitary vibratome sections from a *Sox2^CreERT2/+^; R26^EYFP/+^; Drd2*^−^*^/^*^−^ mouse. Nuclei are stained with TOPRO3 (blue). Start of the lineage tracing by TAM injection occurred *before* **(B)** or *during* **(C)** tumor development. Double SOX2^+^/YFP^+^ are indicated (arrows). Some lineage-traced (YFP^+^) cells do not contain SOX2 anymore (arrowheads). AP, anterior pituitary; IL, intermediate lobe. Scale bar = 50 µm. **(D)** Immunofluorescence staining for prolactin (PRL) (red) and YFP (green) in pituitary vibratome sections from a SOX2^+^ lineage tracing control mouse (i.e., *Sox2^CreERT2/+^; R26^EYFP/+^; Drd2^+/+^*, i.e., without pituitary tumor formation) and a SOX2^+^ lineage tracing *Drd2*^−^*^/^*^−^ mouse (i.e., *Sox2^CreERT2/+^*; *R26^EYFP/+^*; *Drd2*^−^*^/^*^−^, i.e., with pituitary tumor formation). Nuclei are stained with TOPRO3 (blue). Start of the lineage tracing by TAM injection occurred *before* tumor development [see **(A)**]. Boxed regions are magnified and shown as overlay of the three colors (upper panels), or of YFP^+^ (green) and PRL^+^ (red) signals (lower panels). Some cells with co-localized YFP^+^ (green) and PRL^+^ (red) signals are pointed to (arrows). Some cells with only YFP^+^ signal and no PRL^+^ signal are also indicated (arrowheads). AP, anterior pituitary; IL, intermediate lobe; PP, posterior pituitary. Scale bar = 50 µm. **(E)** Immunofluorescence staining for PRL (red) and YFP (green) in pituitary vibratome sections from a SOX2^+^ lineage tracing control mouse (i.e., *Sox2^CreERT2/+^; R26^EYFP/+^; Drd2^+/+^*, i.e., without pituitary tumor formation) and a SOX2^+^ lineage tracing *Drd2*^−^*^/^*^−^ mouse (i.e., *Sox2^CreERT2/+^; R26^EYFP/+^; Drd2*^−^*^/^*^−^, i.e., with pituitary tumor formation). Nuclei are stained with TOPRO3 (blue). Start of the lineage tracing by TAM injection occurred *during* tumor growth [i.e., at 6 months of age; see (**A**)]. Boxed regions are magnified and shown as overlay of the three colors (upper panels), or of YFP^+^ (green) and PRL^+^ (red) signals (lower panels). Some cells with co-localized YFP^+^ (green) and PRL^+^ (red) signals are pointed to (arrows). Some cells with only YFP^+^ signal and no PRL^+^ signal are also indicated (arrowheads). AP, anterior pituitary; IL, intermediate lobe; PP, posterior pituitary. Scale bar = 50 µm.

The majority of the pituitary tumors occur in the AP, the major endocrine part of the gland. Transgenic overexpression of pituitary tumor-transforming gene (PTTG), the mammalian index securin, in early endocrine pituitary progenitor cells (using the promoter of αGSU, the α − subunit of the glycoprotein hormones TSH, LH, and FSH) leads to the eventual development of uni- or pluri-hormonal microadenomas (expressing LH alone, or LH with TSH and GH, respectively). These findings suggest that early pituitary progenitor cells have the capacity to directly give rise to tumors, although a paracrine impact was not excluded (37) (Table 1). Transgenic overexpression of the pituitary stem cell marker PROP1 under control of the αGSU promoter also leads (after 1 year) to pituitary tumors including non-hormonal, GH-, PRL-, GH/PRL-, PRL/αGSU-, and TSH-producing adenomas (38, 39). PROP1 overexpression was not a feature of the adenomas but was detected in the non-neoplastic regions surrounding them, again suggesting a paracrine tumor-stimulatory role of the mutant (stem/progenitor) cells (Table 1). Ectopic overexpression of the pituitary stem cell protein BMI1 (45) caused after 1 year the development of both IL and AP tumors that were positive for ACTH and β-endorphin (40). Finally, our group recently performed SOX2^+^ lineage tracing in the *Drd2^−/−^* mouse model developing prolactinomas in the AP. The SOX2^+^ cell progeny (as indicated by “yellow fluorescent protein” or YFP^+^ signal) increased in abundance during tumor development and growth, but co-localization of YFP and PRL was low, indicating that the large majority of the tumor (PRL^+^) cells did not descend from the SOX2^+^ (stem) cells (Figure 1).

Taken together, recent studies provide arguments that the pituitary stem cells may be involved in the process of tumor development and growth in the gland. The stem cell activation, as, for instance, due to genetic mutations in the cells, may lead to paracrine stimulation of the proliferative activity of neighboring cells. The stem cell activation may also represent a defense reaction to the tumorigenic event occurring in the other cell population(s) in the gland, which may paradoxically feed the tumor by paracrine influences. On the other hand, pituitary tumors (or at least certain histotypes) may directly descend from (mutated) tissue stem cells as shown in tumors of other tissues (13–18). Lineage tracing of the pituitary stem cells in mouse models developing pituitary tumors are essential to unravel the nature of the link between the tissue’s stem cells and tumorigenesis (and the driving TSC) (Table 1).

## Aberrant Activity of Stem Cell Regulatory Pathways in Pituitary Tumors

Given their long-lived self-renewal potency, tissue stem cells must be kept innocuous by strict regulatory control. From the studies described above, pituitary stem cells appear to reside in an activated state during tumorigenesis in the gland. This activation may be due to, or associated with, aberrant activity of the signaling pathways regulating the stem cells. Knowledge on aberrant functioning of these pathways in pituitary tumorigenesis is scarce. Here, we summarize what is known about the major stem cell signaling pathways (i.e., NOTCH, WNT, EMT, SHH, Hippo, and epidermal growth factor-EGF/FGF) in pituitary tumors, and where investigations have been conducted, in the candidate TSC populations (Table 1).

The NOTCH pathway can be either activated or suppressed depending on pituitary tumor histotype (46). For instance, *NOTCH3* and *JAG1* expression was found upregulated in NFPA in comparison to normal human pituitary tissue (47–49). Strangely, the NOTCH target gene *HES1* was found downregulated (48). In prolactinomas, an increase in *NOTCH3* was described (50), although not confirmed in another study (48). Regarding the proposed pituitary tumor TSC populations, knowledge so far only involves expression analysis and support for a functional role is still missing (Table 1). Pronounced gene expression of NOTCH pathway components (i.e., *DLL1, JAG1, HES1, NOTCH1*, and *NOTCH2*) was found in the human pituitary tumor SP (28) while other candidate TSC populations (i.e., human tumorspheres and CD133^+^ cell populations) showed increased expression of *DLL1, JAG2, NOTCH1*, and *NOTCH4* (25, 26).

Supporting the potential involvement of the WNT pathway in pituitary tumorigenesis, upregulated gene and protein expression of WNT4 has been detected in somatotropinomas, prolactinomas, and thyrotropinomas as compared to normal human pituitary tissue (51, 52). The WNT target gene *PITX2* was found increased in NFPA and an anti-apoptotic role was suggested (53, 54). Human pituitary adenomas showed upregulated gene expression of WNT pathway components (e.g., *LEF1, LGR5, TCF7L1, WNT5A*) in the SP compared to the predominant non-SP cells (28) (Table 1). As described above, the ACP mouse model showed that WNT activation in the pituitary’s resident stem cells is a key event in ACP tumor development and growth, and that the mutated SOX2^+^ cells promote the proliferation of the surrounding cells, probably *via* paracrine signaling that also includes several WNT ligands showing upregulated gene expression (e.g., *Wnt5a, Wnt6, Wnt10a*) (3, 4, 32). Consistent with this, human ACP also shows enhanced expression of canonical WNT target genes (e.g., *LEF1, AXIN2, PITX2*) in comparison to normal pituitary tissue (55, 56). By contrast, upregulation of WNT signaling was not observed in human PCP (5, 56, 57).

EMT plays a central role in cancer pathogenesis, including invasion and therapy resistance (58, 59), and may promote the induction and function of CSC (60–62). The process implies the downregulation of the cell–cell adhesion molecule E-cadherin. As analyzed in somatotropinomas, decreased expression of E-cadherin may be associated with enlarged tumors and higher invasiveness, and reduced susceptibility to pharmacotherapy (63). Transcriptomic profiling of human pituitary tumor SP supported the occurrence of EMT, at least to a certain level (known as partial EMT), in the candidate TSC population (28) (Table 1). In comparison to the non-SP cells, there was a downregulation of epithelial markers (*CDH1, CLDN1*) and a corresponding increase in mesenchymal markers (*FN1, VIM*) as well as EMT mediators (*SNAI1, SNAI2, ZEB1, ZEB2*) and regulators (e.g., *CXCR4, FOSL2, FOXC1, LEF1, MMP1*) (28).

SHH activity may also differ according to pituitary adenoma histotype. Whereas SHH pathway components are downregulated in NFPA in comparison to normal human pituitary tissue, *GLI1* was found to be overexpressed in somatotropinomas and corticotropinomas (49, 64). GLI1 protein expression appears to follow SOX2 expression in a number of human pituitary adenomas (65). SHH signaling may also be activated in human ACP given the upregulated expression of several pathway components (*GLI1, GLI2, GLI3, PTCH1*, and *SHH*) (32, 55, 56, 66), not observed in PCP (56, 57). Transgenically mutant β-catenin-induced ACP in mice showed increased production of SHH by the mutated SOX2^+^ cells (4, 32) (Table 1).

The Hippo pathway is important in determining growth and size of organs (67, 68). Downregulation of the Hippo pathway with consequent elevation of YAP/TAZ levels has been found in several cancers, suggesting that Hippo signaling may exert a tumor-suppressive function (69). The role of Hippo signaling in pituitary tumorigenesis is unexplored territory; elevated gene expression of the Hippo pathway components *LATS2, TEAD4*, and *YAP1* and of their downstream targets *CTGF* and *CYR61* has recently been reported in the SP of human pituitary adenomas (28) (Table 1).

Research using human pituitary adenomas and some invasive pituitary tumors has revealed expression of EGF and/or EGFR (70–76). FGFs are also elevated in pituitary adenomas as compared to normal human pituitary (72). Upregulated gene expression of cognate receptors of these growth factors, i.e., *EGFR, FGFR1*, and *FGFR3*, was detected in the human pituitary tumor SP (28), suggesting that EGF/FGF signaling may function to drive cell proliferation in these candidate TSC (Table 1).

Taken together, information on stem cell signaling pathways in pituitary tumorigenesis is presently very limited. Investigations are needed to clarify whether aberrant activity of pathways, such as NOTCH, WNT, EMT, SHH, Hippo, and EGF/FGF, is associated with tumorigenesis, for instance, whether their deregulation in the pituitary resident stem cells leads to the generation of TSC that drive tumor growth, or whether the deregulation promotes tumorigenesis through paracrine signaling between the activated tissue stem cells and surrounding tumor cells. These examinations may reveal valuable therapeutic targets and lead to new approaches for treating pituitary tumors.

## Conclusion and Perspective

Cells of stemness nature may occupy an important position in the pituitary tumorigenesis process. Candidate TSC have been spotlighted that may drive tumor growth and therapy resistance, eventually leading to re-growth. The pituitary’s own stem cells appear activated during tumorigenesis in the gland, potentially associated with altered activity of stem cell regulatory pathways. Whether there is a link between the tissue’s stem cells and the TSC, is not clear yet. Several of the recent studies lean support to a paracrine-inducing and -supporting role of the resident stem cells, rather than to a direct lineal relationship with the tumor (and its TSC). However, available studies are still scarce and current pituitary tumor mouse models do not highly recapitulate the human disease. Many more stem cell lineage tracing studies are needed to convincingly unravel the stem cell link(s), which also may be different according to pituitary tumor type. Alternatively, specific ablation of the stem cells [e.g., using a controllable transgenic system (41–43)] would unravel whether these cells are (in-)dispensable for pituitary tumorigenesis, regardless of their direct or indirect involvement. Future research may, thus, adhere to the following cycle: (1) further unraveling the genetic and molecular grounds of pituitary tumorigenesis, preferably in humans using tumor samples; (2) developing (more) appropriate pituitary tumor study models from these new insights, in particular genetically modified mouse models; (3) scrutinizing stem cell behavior, and specifically stem cell lineage tracing, in the mouse models obtained; and (4) re-applying the stem cell-associated results (like markers and pathways) to human tumors. This cycling has recently been best exemplified for ACP (3–5, 32), and serious efforts are now needed for all other pituitary tumor types. Meanwhile, the behavior of the stem cells can already be analyzed in the pituitary tumor mouse models available (77) and molecular mechanisms underlying stem cell activation (if any) elucidated. Further unraveling the position of the stem cells and the potential TSC, and their (de-)regulation, will not only provide deeper fundamental insight in the as yet poorly understood mechanisms of pituitary tumorigenesis but also clues toward novel drug targets and the development of improved therapies, particularly toward prevention of relapse and treatment of resistant tumors. It is anticipated that transcriptomic profiling of the candidate TSC and the activated tissue stem cells will reveal signaling networks that drive the (enhanced) activity of the tumor/tissue stem cells. Those “big data” may serve as a valuable resource to identify potentially interesting (novel) disease biomarkers and therapeutic targets for further examination. Lineage tracing of the tissue stem cells, and scrutiny of the molecular profile of isolated populations of stem cells such as the SP discussed here will provide an extremely valuable platform upon which to build these research investigations and ultimately identify suitable molecular targeted therapies to translate into a clinical setting. Thus, stem cells represent interesting candidates for a leading role on the pituitary tumorigenesis scene, which eventually may be transposed to clinical scenarios.

## Ethics Statement

Animal experiments were approved by the KU Leuven Ethical Committee.

## Author Contributions

HV compiled the literature data and wrote the manuscript. HR compiled the data for part of the review, and provided the figure for which she performed the experiments.

## Conflict of Interest Statement

The authors declare that the research was conducted in the absence of any commercial or financial relationships that could be construed as a potential conflict of interest.
